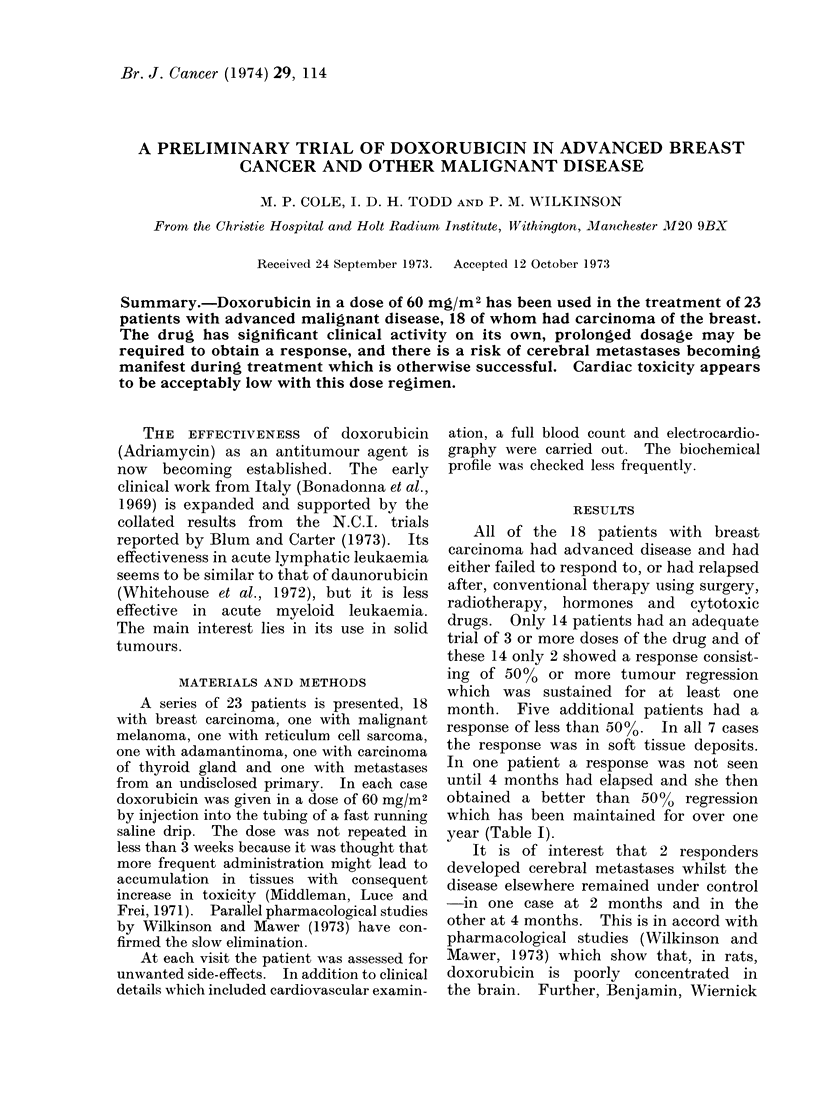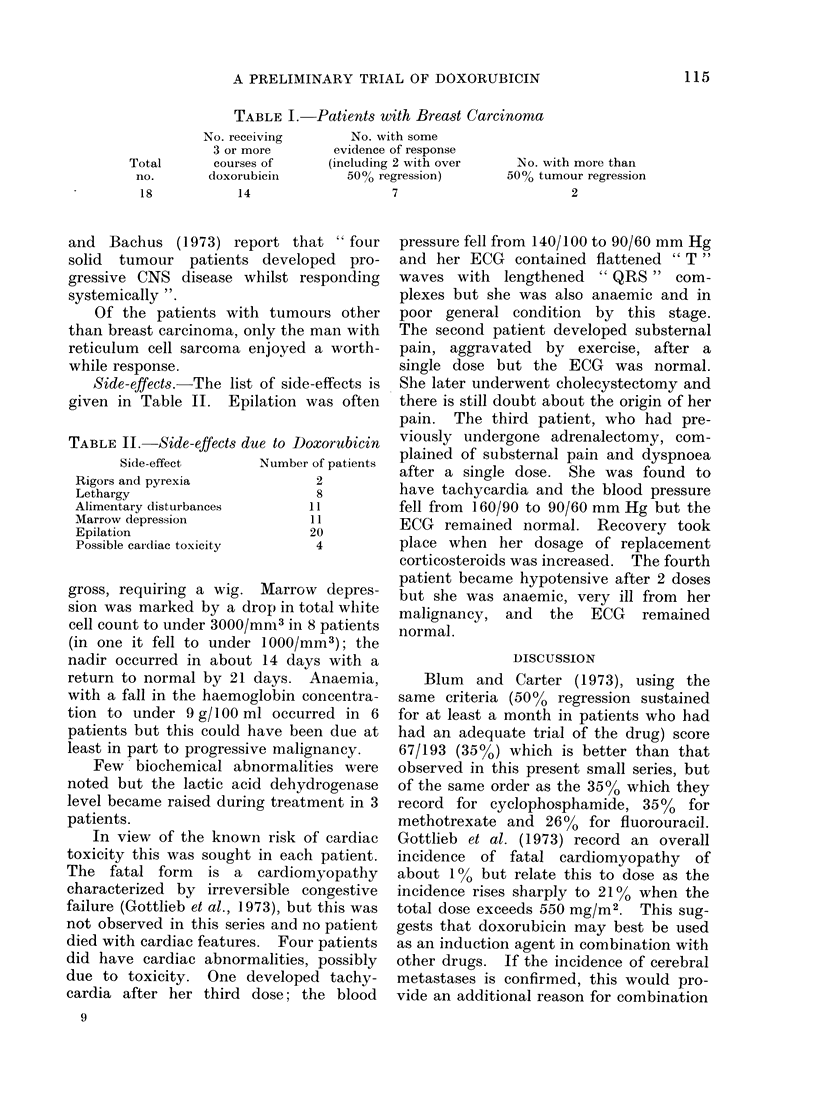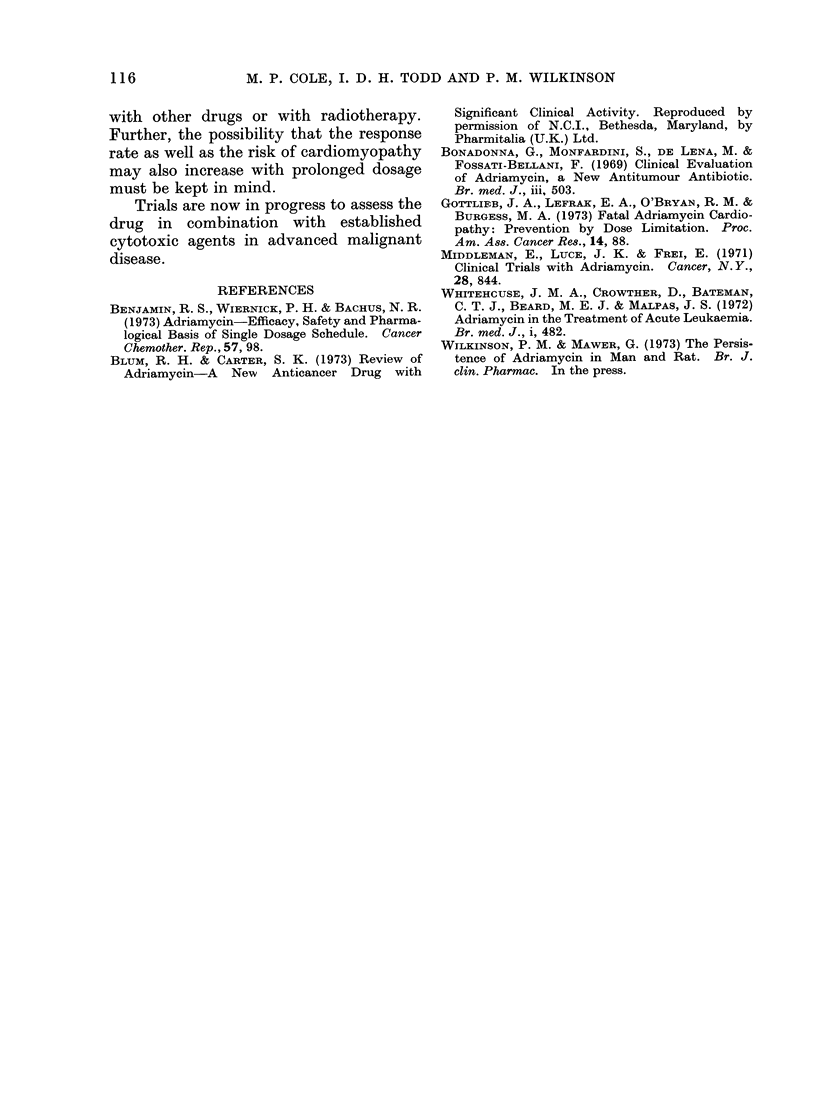# A Preliminary Trial of Doxorubicin in Advanced Breast Cancer and Other Malignant Disease

**DOI:** 10.1038/bjc.1974.47

**Published:** 1974-02

**Authors:** M. P. Cole, I. D. H. Todd, P. M. Wilkinson

## Abstract

Doxorubicin in a dose of 60 mg/m^2^ has been used in the treatment of 23 patients with advanced malignant disease, 18 of whom had carcinoma of the breast. The drug has significant clinical activity on its own, prolonged dosage may be required to obtain a response, and there is a risk of cerebral metastases becoming manifest during treatment which is otherwise successful. Cardiac toxicity appears to be acceptably low with this dose regimen.


*Br. J. Cancer* (1974) **29**, 114

### A PRELIMINARY TRIAL OF DOXORUBICIN IN ADVANCED BREAST CANCER AND OTHER MALIGNANT DISEASE

M. P. COLE, I. D. H. TODD AND P. M. WILKINSON

*From the Christie Hospital and Holt Radium Institute, Withington, Manchester M20 9BX*

Received 24 September 1973. Accepted 12 October 1973

**Summary.—Doxorubicin in a dose of 60 mg/m$^2$ has been used in the treatment of 23 patients with advanced malignant disease, 18 of whom had carcinoma of the breast. The drug has significant clinical activity on its own, prolonged dosage may be required to obtain a response, and there is a risk of cerebral metastases becoming manifest during treatment which is otherwise successful. Cardiac toxicity appears to be acceptably low with this dose regimen.**

THE EFFECTIVENESS of doxorubicin (Adriamycin) as an antitumour agent is now becoming established. The early clinical work from Italy (Bonadonna *et al.*, 1969) is expanded and supported by the collated results from the N.C.I. trials reported by Blum and Carter (1973). Its effectiveness in acute lymphatic leukaemia seems to be similar to that of daunorubicin (Whitehouse *et al.*, 1972), but it is less effective in acute myeloid leukaemia. The main interest lies in its use in solid tumours.

### MATERIALS AND METHODS

A series of 23 patients is presented, 18 with breast carcinoma, one with malignant melanoma, one with reticulum cell sarcoma, one with adamantinoma, one with carcinoma of thyroid gland and one with metastases from an undisclosed primary. In each case doxorubicin was given in a dose of 60 mg/m$^2$ by injection into the tubing of a fast running saline drip. The dose was not repeated in less than 3 weeks because it was thought that more frequent administration might lead to accumulation in tissues with consequent increase in toxicity (Middleman, Luce and Frei, 1971). Parallel pharmacological studies by Wilkinson and Mawer (1973) have confirmed the slow elimination.

At each visit the patient was assessed for unwanted side-effects. In addition to clinical details which included cardiovascular examination, a full blood count and electrocardiography were carried out. The biochemical profile was checked less frequently.

### RESULTS

All of the 18 patients with breast carcinoma had advanced disease and had either failed to respond to, or had relapsed after, conventional therapy using surgery, radiotherapy, hormones and cytotoxic drugs. Only 14 patients had an adequate trial of 3 or more doses of the drug and of these 14 only 2 showed a response consisting of 50% or more tumour regression which was sustained for at least one month. Five additional patients had a response of less than 50%. In all 7 cases the response was in soft tissue deposits. In one patient a response was not seen until 4 months had elapsed and she then obtained a better than 50% regression which has been maintained for over one year (Table I).

It is of interest that 2 responders developed cerebral metastases whilst the disease elsewhere remained under control —in one case at 2 months and in the other at 4 months. This is in accord with pharmacological studies (Wilkinson and Mawer, 1973) which show that, in rats, doxorubicin is poorly concentrated in the brain. Further, Benjamin, Wiernick

TABLE I.—*Patients with Breast Carcinoma*

| Total no. | No. receiving 3 or more courses of doxorubicin | No. with some evidence of response (including 2 with over 50% regression) | No. with more than 50% tumour regression |
|---|---|---|---|
| 18 | 14 | 7 | 2 |

and Bachus (1973) report that "four solid tumour patients developed progressive CNS disease whilst responding systemically".

Of the patients with tumours other than breast carcinoma, only the man with reticulum cell sarcoma enjoyed a worthwhile response.

*Side-effects.*—The list of side-effects is given in Table II. Epilation was often gross, requiring a wig. Marrow depression was marked by a drop in total white cell count to under $3000/mm^3$ in 8 patients (in one it fell to under $1000/mm^3$); the nadir occurred in about 14 days with a return to normal by 21 days. Anaemia, with a fall in the haemoglobin concentration to under 9 g/100 ml occurred in 6 patients but this could have been due at least in part to progressive malignancy.

TABLE II.—*Side-effects due to Doxorubicin*

| Side-effect | Number of patients |
|---|---|
| Rigors and pyrexia | 2 |
| Lethargy | 8 |
| Alimentary disturbances | 11 |
| Marrow depression | 11 |
| Epilation | 20 |
| Possible cardiac toxicity | 4 |

Few biochemical abnormalities were noted but the lactic acid dehydrogenase level became raised during treatment in 3 patients.

In view of the known risk of cardiac toxicity this was sought in each patient. The fatal form is a cardiomyopathy characterized by irreversible congestive failure (Gottlieb *et al.*, 1973), but this was not observed in this series and no patient died with cardiac features. Four patients did have cardiac abnormalities, possibly due to toxicity. One developed tachycardia after her third dose; the blood pressure fell from 140/100 to 90/60 mm Hg and her ECG contained flattened "T" waves with lengthened "QRS" complexes but she was also anaemic and in poor general condition by this stage. The second patient developed substernal pain, aggravated by exercise, after a single dose but the ECG was normal. She later underwent cholecystectomy and there is still doubt about the origin of her pain. The third patient, who had previously undergone adrenalectomy, complained of substernal pain and dyspnoea after a single dose. She was found to have tachycardia and the blood pressure fell from 160/90 to 90/60 mm Hg but the ECG remained normal. Recovery took place when her dosage of replacement corticosteroids was increased. The fourth patient became hypotensive after 2 doses but she was anaemic, very ill from her malignancy, and the ECG remained normal.

### DISCUSSION

Blum and Carter (1973), using the same criteria (50% regression sustained for at least a month in patients who had had an adequate trial of the drug) score 67/193 (35%) which is better than that observed in this present small series, but of the same order as the 35% which they record for cyclophosphamide, 35% for methotrexate and 26% for fluorouracil. Gottlieb *et al.* (1973) record an overall incidence of fatal cardiomyopathy of about 1% but relate this to dose as the incidence rises sharply to 21% when the total dose exceeds $550 mg/m^2$. This suggests that doxorubicin may best be used as an induction agent in combination with other drugs. If the incidence of cerebral metastases is confirmed, this would provide an additional reason for combination with other drugs or with radiotherapy. Further, the possibility that the response rate as well as the risk of cardiomyopathy may also increase with prolonged dosage must be kept in mind.

Trials are now in progress to assess the drug in combination with established cytotoxic agents in advanced malignant disease.

### REFERENCES

BENJAMIN, R. S., WIERNICK, P. H. & BACHUS, N. R. (1973) Adriamycin—Efficacy, Safety and Pharmacological Basis of Single Dosage Schedule. *Cancer Chemother. Rep.*, **57**, 98.

BLUM, R. H. & CARTER, S. K. (1973) Review of Adriamycin—A New Anticancer Drug with Significant Clinical Activity. Reproduced by permission of N.C.I., Bethesda, Maryland, by Pharmitalia (U.K.) Ltd.

BONADONNA, G., MONFARDINI, S., DE LENA, M. & FOSSATI-BELLANI, F. (1969) Clinical Evaluation of Adriamycin, a New Antitumour Antibiotic. *Br. med. J.*, iii, 503.

GOTTLIEB, J. A., LEFRAK, E. A., O'BRYAN, R. M. & BURGESS, M. A. (1973) Fatal Adriamycin Cardiopathy: Prevention by Dose Limitation. *Proc. Am. Ass. Cancer Res.*, **14**, 88.

MIDDLEMAN, E., LUCE, J. K. & FREI, E. (1971) Clinical Trials with Adriamycin. *Cancer, N.Y.*, **28**, 844.

WHITEHCUSE, J. M. A., CROWTHER, D., BATEMAN, C. T. J., BEARD, M. E. J. & MALPAS, J. S. (1972) Adriamycin in the Treatment of Acute Leukaemia. *Br. med. J.*, i, 482.

WILKINSON, P. M. & MAWER, G. (1973) The Persistence of Adriamycin in Man and Rat. *Br. J. clin. Pharmac.* In the press.